# Estimating accuracy of RNA-Seq and microarrays with proteomics

**DOI:** 10.1186/1471-2164-10-161

**Published:** 2009-04-16

**Authors:** Xing Fu, Ning Fu, Song Guo, Zheng Yan, Ying Xu, Hao Hu, Corinna Menzel, Wei Chen, Yixue Li, Rong Zeng, Philipp Khaitovich

**Affiliations:** 1 Key lab of Systems Biology, Shanghai Institutes for Biological Sciences, China Academy of Sciences, Shanghai, 200031, PR China; 2 Partner Institute for Computational Biology, 320 Yue Yang Road, Shanghai, 200031, PR China; 3 Max Planck Institute for Molecular Genetics, Ihnestrasse 63-73, D-14195 Berlin, Germany; 4 Max-Planck-Institute for Evolutionary Anthropology, Deutscher Platz 6, D-04103 Leipzig, Germany

## Abstract

**Background:**

Microarrays revolutionized biological research by enabling gene expression comparisons on a transcriptome-wide scale. Microarrays, however, do not estimate absolute expression level accurately. At present, high throughput sequencing is emerging as an alternative methodology for transcriptome studies. Although free of many limitations imposed by microarray design, its potential to estimate absolute transcript levels is unknown.

**Results:**

In this study, we evaluate relative accuracy of microarrays and transcriptome sequencing (RNA-Seq) using third methodology: proteomics. We find that RNA-Seq provides a better estimate of absolute expression levels.

**Conclusion:**

Our result shows that in terms of overall technical performance, RNA-Seq is the technique of choice for studies that require accurate estimation of absolute transcript levels.

## Background

The ability to measure messenger RNA (mRNA) expression levels of thousands of genes simultaneously gave an enormous boost to biological research since the introduction of microarrays approximately 10 years ago. Microarrays, however, are designed for comparative studies and provide only limited information about absolute gene expression levels [1,2]. This limitation comes from differences in hybridization efficiency, as well as differences in cross-hybridization background among millions of array probes and is difficult to account for. This limitation, however, is largely negligible for comparative, rather than absolute, expression level analyses, explaining the enormous utility of microarrays for a large spectrum of biological studies. Still, accurate estimation of absolute transcript levels is central to a number of applications. Technically, it would allow combining mRNA expression measurements produced by different platforms [3-5]. Biologically, knowledge of absolute transcript levels within cells and tissues would allow direct comparison to other measurements from the same biological system, thus providing a basis for systematic evaluation and modelling of regulatory processes [6-8]. Another important area of application is splicing. In humans, as well as in other species, a great proportion of transcriptome complexity is thought to arise through alternative splicing of exons within a single genomic locus. In humans, for instance, at least 47% of genes show evidence of alternative splicing with nearly 3 isoforms per gene, on average [9]. Currently, however, identification and quantification of individual transcriptional isoforms is a major challenge. Accurate estimation of absolute expression levels of individual exons and exon junctions would greatly facilitate reconstruction of all transcript isoforms simultaneously present in the samples studied [10,11].

In the last few years, several novel high-throughput sequencing technologies producing millions of sequences per single sequencing run have emerged [12-15]. One application of these technologies is transcriptome sequencing, also known as RNA-Seq [13,16,17]. Such an approach has several advantages over microarray technology, including the ability to detect novel transcripts and transcript isoforms, distinguish between closely related paralogous sequences, and quantify expression in a "digital" rather than "analog" manner [13,16-18]. It remains unclear, however, whether RNA-Seq can provide accurate estimates of absolute transcript levels. Previous studies have shown that sequencing reads density tends to vary along the length of a transcript – an observation that indicates RNA-Seq is not bias-free [13,16]. Biases, such as preferential selection/exclusion of certain sequences, could potentially take place during adapter ligation step, PCR amplification, and/or sequencing itself. In fact, differences in ligation efficiency have been already demonstrated in high-throughput sequencing experiments [19,20]. Still, the effect these biases may have on estimation of the absolute transcript levels is currently unknown. Several recent studies have compared transcript expression levels measured in human and mouse samples using both conventional microarrays and RNA-Seq [13,16]. In all cases the expression levels showed good agreement between the two technologies with correlations ranging from 0.62 to 0.75. Still, correlation between the methods is lower than the correlation between technical replicates within each method (average, *r *= 0.96), leaving a large proportion of differences between the methods unexplained. In this study, we use gene expression levels measured using a third technology – shotgun mass spectroscopy – to assess the relative accuracy of the two transcriptome quantification approaches with respect to absolute transcript level measurements.

## Results and Discussion

Here, we measure absolute gene expression levels in human brain samples using three different methodologies: Affymetrix gene microarrays, high throughput sequencing (Illumina, formerly Solexa), and mass spectrometry-based label-free proteomics. Among different brain regions, we have chosen cerebellar cortex due to its relative histological homogeneity, facilitating the dissection procedure and reducing biological variation due to tissue heterogeneity [21]. All cerebellar tissues used in this study came from individuals that suffered sudden death for reasons unrelated to the brain, and mRNA quality was high and comparable across all samples (see Additional file 1: Table S1).

### mRNA measurements by arrays and sequencing

We first determined whether we could reproduce the agreement between mRNA expression estimates measured by microarrays and by RNA-Seq reported in other studies. For this purpose, we collected mRNA expression data in two independent cerebellar samples, each containing pooled mRNA from 5 adult human individuals, using both methodologies (Methods). None of the individuals was shared between the two pools. Using Affymetrix Human Exon 1.0 ST Arrays, we found 8,717 and 6,444 genes with mRNA expression above the detection threshold in the first and the second pooled samples, respectively (Methods). Out of these genes, the vast majority (6,424) was expressed in both samples. Further, gene expression values in the two samples were highly correlated (Person correlation, *r *= 0.95, *p *< 2.2e-16) (see Additional file 1: Figure S1). For RNA-Seq, we sequenced each of the two pooled samples twice, resulting in a total of 5,067,363 sequence reads that could be mapped to the human genome (Methods). In this dataset, 13,582 out of 21,541 annotated known protein-coding genes (Ensembl release 49) were represented by at least two independent sequences and 5,724 by at least 20 (Methods). Although the total number of sequences differed among the four sequencing experiments (see Additional file 1: Table S2), gene expression levels estimated by sequence coverage showed high positive correlation between both the biological and the technical replicates (see Additional file 1: Figure S2). Thus, in agreement with previously published studies, gene expression measurements show relatively little variation within each method [13,16,17].

**Figure 1:**
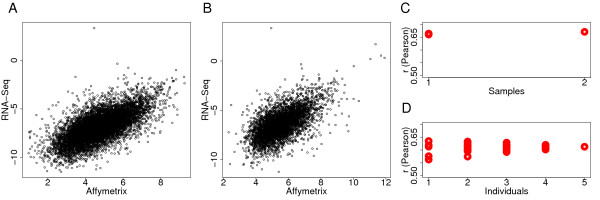
**Correlation between mRNA expression levels measured by Affymetrix microarrays and RNA-Seq**. mRNA expression levels measured by RNA-Seq in two pooled samples of 5 individuals and by microarrays in the same samples (A) or in 5 independent individual samples (B). Shown are expression levels of 8,441 and 4,758 genes, respectively, expressed above background on at least one of the microarrays in a given experiment and represented by at least two independent sequence reads in RNA-Seq (see Methods for details). (C and D) Person correlation coefficients (*r*) from comparisons between RNA-Seq and microarray measurements based on each microarray separately and on average expression from all possible microarray combinations for two pooled and 5 individual samples, respectively (see Additional file 1: Table S3 for details).

In further agreement with previous observations [13,16], we find reasonably good positive correlation between gene expression levels estimated by the two methods. Namely, we observe Person correlation's *r *= 0.67 (*p *< 2.2e-16) in a set of 8,441 genes with mRNA expression detected above background in at least one of the two samples by both techniques (Figure 1A and 1C). The strength of the correlation was similar when the two samples were considered individually (*r *= 0.66 for both samples, Figure 1C and Additional file 1: Table S3). Further, the strength of correlation did not depend much on the sequence coverage and the array detection cutoff, or on the type of correlation test used (see Additional file 1: Figure S3 and Table S3).

Next, to test whether biological variation among samples would substantially reduce correlation strength, we compared expression levels determined by RNA-Seq in two pooled samples to the microarray data obtained from different individuals. For this purpose we used expression measurements obtained using Affymetrix Exon Arrays in 5 individual adult human cerebellar samples, none of which were included in the two pooled samples (see Additional file 1: Table S1). Using these data, we find that correlations between microarray and RNA-Seq expression measurements were reduced only slightly, both for the average expression of the 5 individuals (Person correlation *r *= 0.61, *p *< 2.2e-16) and for each of the individual measurements (Figure 1B and 1D). In general, since individual measurements from all 5 samples were highly correlated, combining any number of individuals did not influence the result (Figure 1D and Additional file 1: Table S3). Thus, individual variation among adult human cerebellar samples did not have much influence on the correlation between microarray and RNA-Seq measurements.

### Assessing mRNA measurements accuracy with proteomics data

Despite the observed agreement between microarray and RNA-Seq expression measurements, the correlation is not perfect leaving a relatively large proportion of total expression variation (from 48% in [13] to 57% in our data) unexplained. In order to evaluate which methodology provides better estimates of absolute mRNA expression levels, we compared the two sets of mRNA expression measurements to a third dataset: protein expression data from adult human cerebellum. Protein data were collected using 2D-LC MS/MS from four individual samples, each with two experimental replicates (see Additional file 1: Table S1). In these data, we could identify 179,875 peptides corresponding to 1,577 genes with peptide identification FDR set to 0.5% (Methods). Out these genes, 1,037 represented by at least two peptides were included in the following analysis.

Biologically, mRNA and protein expression levels cannot be expected to correlate perfectly due to post-transcriptional regulation. Nonetheless, positive correlation between protein and mRNA expression levels has previously been shown in a variety of systems from bacteria to mammals, with correlation coefficients ranging from 0.2 to 0.5 [6,22-27], thus indicating that mRNA and protein expression levels are not fully independent. Further, since technical and stochastic variation are extremely unlikely to result in better correlation between mRNA and protein expression measurements, we argue that the technology resulting in better correlation must provide more accurate measurements.

**Figure 2:**
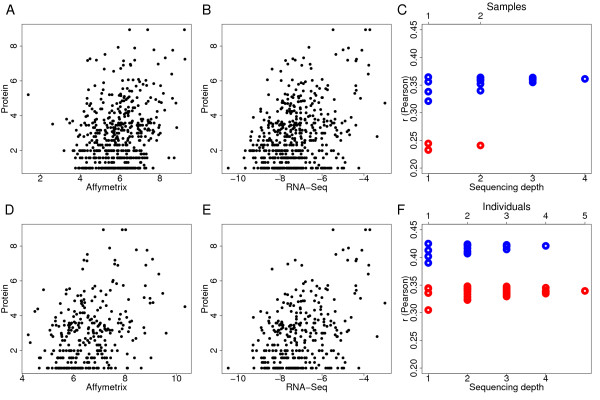
**Correlation between protein and mRNA expression levels measured by Affymetrix arrays and RNA-Seq**. Protein expression was measured in four individual samples with technical replicates. mRNA expression was measured by microarrays and RNA-Seq in two pooled samples (A, B, and E), and by microarrays in 5 individual samples (D). Shown are expression intensities of 520 (A and B) and 306 (D and E) genes expressed above background on all microarrays in a given experiment and represented by at least twenty independent sequence reads in RNA-Seq. (Cand F) Person correlation coefficients (*r*) from comparisons between RNA-Seq and protein measurements (blue) and between microarray and protein measurements (red) for two pooled samples and 5 individual samples, respectively. For RNA-Seq data, the correlations were based on each sequencing experiment separately and on average expression from all possible experiment combinations. For microarrays, the correlations were based on expression values from each microarray separately and on average expression from all possible microarray combinations (see Additional file 1: Table S4 for details).

In agreement with previous results, we find only moderate correlation between protein and mRNA expression levels when using microarray measurements (Pearson correlation, *r *= 0.24, *p *= 2.7e-8, *N *= 520). Using RNA-Seq expression measurements for the same set of genes, we find substantially higher, albeit moderate, correlation (Pearson correlation, *r *= 0.36, *p *< 2.2e-16, *N *= 520). The difference between the two mRNA quantification methodologies was significant (*p *< 0.05) and consistent for both samples, as well as for their average, and did not depend on the sequence coverage depth, detection cutoff, or type of the correlation test used (Figure 2C and Additional file 1: Table S4). Further, using microarray data from five individual samples instead of the two pooled samples gave similar results (Pearson correlation, *r *= 0.34, *p *= 1.1e-9 and *r *= 0.42, *p *= 1.5e-14 for microarray and RNA-Seq, respectively;*N *= 306). Again, correlation strength between protein and microarray measurements, as well as that between protein and RNA-Seq, was consistent among all samples and did not depend on the sequence coverage depth, detection cutoff, or type of the correlation test used (Figure 2F and Additional file 1: Table S4). Notably, we consistently find better correlation between mRNA and protein data using RNA-Seq measurements, even though the same four individuals were used for both proteomics and microarray measurements (see Additional file 1: Table S1). Thus, individual or technical variations do not explain the better agreement between RNA-Seq and protein expression measurements among samples used in this study. Finally, excluding all exons shared by multiple isoforms and calculating transcript expression using the highest level or the mean of all isoforms did not change the result (see Additional file 1: Figure S4). Taken together, our results indicate that, following standard microarray and RNA-Seq methodology, RNA-Seq provides a better estimate of observed protein levels than microarrays.

**Figure 3:**
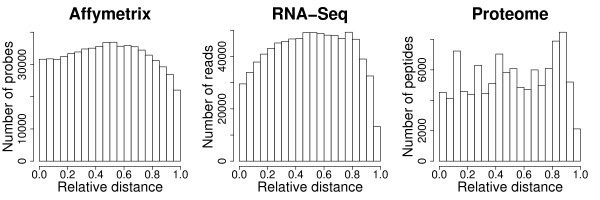
**Distribution of expression signals within genes for the three methodologies**. The histograms show total signal density for 6424, 13582, and 1577 genes with detectable expression estimated by microarrays, RNA-Seq, or proteomics, respectively. In order to account for length differences among genes, for each gene we normalized the distances between the middle positions of the detected expression measurements (array probes, sequence reads, or peptides) and the 5' end of the gene by the gene length.

We note that our method assesses the general accuracy of these two techniques and is not developed as an approach for verifying individual gene expression measurements in a specific experiment. Instead, it demonstrates which technique, RNA-Seq or microarrays, provides more accurate expression estimates as a methodology. Further, our method estimates relative, rather than the absolute accuracies of the two techniques. Using RNA samples with known concentrations spiked into the total RNA samples would provide a more direct way to assess the techniques' accuracy. Still, even though our results are limited to a particular array type and the sample preparation protocols used, they should reflect the general relationship among the three methodologies. In all three techniques, we estimate gene expression levels using standard sample preparation and processing procedures. Further, in all three techniques, an expression signal was calculated over the entire gene length, rather than at a particular transcript part. The Affymetrix microarrays used in this study, Human Exon arrays, contain probes distributed over the entire gene length [28]. In RNA-Seq and shotgun proteomics, measurements are not restricted to predefined probes and, therefore, could potentially detect sequences and peptides corresponding to any location within a gene. Further, for both microarrays and RNA-Seq, we used random primers for the first-strand cDNA synthesis, thus ensuring approximately uniform coverage along the transcripts (Methods). Indeed, analysing the distribution of expression measurements along genes, we find approximately uniform distribution for all three techniques (Figure 3). As this results in a greater total expression signal for longer genes, we used gene expression measures independent of gene length for all three techniques: average sequence coverage for RNA-Seq, average expression level of all detected array probes for microarrays, and average copy number of all detected peptides for proteomics (Methods). Thus, our results should reflect the general relationship between gene expression measurements obtained by the three techniques using comparable and standard procedures.

## Conclusion

In this study, we used protein expression measurements to evaluate the accuracy of two mRNA quantification methods: microarrays and RNA-Seq. Our results show that using standard microarray and RNA-Seq protocols, RNA-Seq provides better estimates of absolute transcript levels. This is particularly encouraging given that the original methodological focus of high throughput sequencing is genomic rather than transcriptomic studies. Thus, methodological adjustments improving accuracy of transcript level estimation by high throughput sequencing might be possible. Our results indicate that RNA-Seq is already the technique of choice for questions relying on accurate absolute transcript level measurements.

## Methods

### Samples

Human tissue was obtained from the NICHD Brain and Tissue Bank for Developmental Disorders at the University of Maryland, Baltimore, MD. The role of the NICHD Brain and Tissue Bank is to distribute tissue, and therefore, cannot endorse the studies performed or the interpretation of results. Informed consent for use of the human tissues for research was obtained in writing from all donors or the next of kin. All subjects were defined as normal controls by forensic pathologists at the NICHD Brain and Tissue Bank. No subjects with prolonged agonal state were used. All samples were taken from the middle part of the cerebellar cortex. No samples showed any detectable RNA degradation, as measured using an Agilent Bioanalyzer (Agilent Technologies, Palo Alto, USA) indicating good tissue preservation. Details of all samples, including age, sex, and RNA quality are given in Table S1.

### RNA preparation and cDNA synthesis

Total RNA was extracted by Trizol reagent (Invitrogen, Carlsbad, CA) according to the manufacturer's instructions and treated for 30 min at 37°C with RNase free DNase I (Ambion, Austin, TX). DNA-free total RNA was purified with the RNeasy MinElute Kit according to the manufacturer's instructions (Qiagen, Valencia, CA). Prior to cDNA synthesis, 10 ug of total RNA was treated with two rounds of RiboMinus kit (Invitrogen) to completely remove the ribosomal RNA.

For the first strand of cDNA synthesis, 2 ug of rRNA-Reduced total RNA was mixed with 500 ng of random primer mix, incubated at 70°C for 5 minutes, and then transferred to an ice bath. The first strand cDNA synthesis was performed according to the standard protocol. Specifically, in reaction mix containing 400 U of Superscript II reverse transcriptase, 75 mM Tris Hcl, pH7.5, 100 mM KCl, 5 mM MgCl2, 0.01 M DTT, and 20 mM dNTPs (Invitrogen) in a total volume of 25 ul; this reaction mix was incubated at 42°C for 60 minutes. The resulting first strand cDNA was used to make second strand cDNA in a reaction mix containing 20 mM dNTPs, 15 U of E. coli DNA Polymerase I and 2 U of E. coli RNase H in a total volume of 100 ul; this reaction mix was incubated at 16°C for 2 hours. The resulting double stranded cDNA was purified using the Qiaquick PCR purification kit (Qiagen). Samples were then fragmented with nebulization technique to yield fragment sizes of 100–300 bps.

### Library preparation for Illumina sequencing

The Illumina library was prepared according to the manufacturer's instructions [29] Specifically, the library was purified with Qiaquick DNA purification kit (Qiagen). The size-selected cDNA was blunt ended with End Repair Enzyme in the presence of 2.5 mM dNTPs (NEB) and 10 mM ATP (Illumina, San Diego, CA). Adenine nucleotide was added to the 3'ends of the blunt ended cDNA with Klenow fragment (3' to 5' exominus) in the presence of 1 mM dATP (NEB) by incubating at 37°C for 30 minutes. The end labeled double stranded cDNA was purified with a Qiaquick DNA purification column (Qiagen). The double stranded cDNA with A-nucleotides on its ends was ligated with adapters (Illumina) using T4 DNA ligase at room temperature for 15 minutes. The samples were then purified with Qiaquick PCR purification kit (Qiagen). Subsequently, the cDNA was amplified with two adapter primers (Illumina) with initial denaturing step at 98°C for 30 seconds, followed by 14 cycles at 98°C for 30 seconds, 65°C for 30 seconds, 72°C for 30 seconds with a final extension cycle at 72°C for 5 minutes. The PCR product was purified with Qiaquick PCR purification kit. The product size of 100–300 bp was gel extracted and used directly for cluster generation and sequencing analysis using Illumina's Solexa Sequencer according to the manufacturer's instructions. All sequences are available at .

### Sequence mapping

To map the resulting 36-nucleotide long sequencing reads to the human genes, we aligned all the reads against the whole genome (hg18) and all the transcripts downloaded from Ensembl database using the SOAP algorithm [30]. We allowed at most two mismatches in each alignment. Reads with multiple "best hit" locations were discarded. We calculated gene expression level as a median number of reads mapped on its isoforms divided by the gene length.

### Microarray processing and analysis

mRNA samples for Affymetrix Human Exon 1.0 ST Arrays were prepared following the standard GeneChip^® ^Whole Transcript (WT) Sense Target Labelling Assay (see manual, P/N 701880) [28]. Prior to expression data analysis, we masked all probes that did not match reference human genomes perfectly (hg18) and did not map to a unique location [31]. To determine whether the signal intensity of a given probe was above the expected level of background noise, we compared the signal intensity for each probe to a distribution of signal intensities of the antigenomic probes with the same GC content. Antigenomic probes are specifically designed by Affymetrix to provide an estimate of the non-specific background hybridization [32]. The probe signal was classified as detected above background if its intensity was larger than the 95% percentile of the background probes with the same GC content [33]. If more than 80% of probes and at least ten probes per transcript were detected, the transcript signal was classified as detected for each individual. To further remove any possible systematic experimental differences between the arrays, we performed a PM-GCBG correction [32] and quantile normalization using R package "affy" [34]. Prior to the normalization, all intensities were base-two-logarithm transformed. The intensities of transcripts were summarized by median polish method. We used the Transcript Cluster Annotations file [28] to map the transcript clusters annotated by Affymetrix to Ensembl genes. In cases where multiple transcript clusters mapped to the same gene, we calculated gene expression as the median of all corresponding transcript clusters. None of the transcript clusters overlapped. All original microarray data is deposited in GEO database [GSE13744].

### Protein sample preparation

Proteins were extracted from 100 mg of frozen cerebellar tissue samples as described elsewhere [35,36] with small modifications. Namely, each individual tissue sample was minced, washed in ice-cold PBS and homogenized in ice-cold lysis buffer (8 M urea, 4% CHAPS, 65 mM DTT, 40 mM Tris, cocktail protease inhibitor, 200 mg of tissue/1 ml) using an electric homogenizer. The resulting protein solution was sonicated on ice for a total of 3 minutes and then centrifugated at 25,000 g for 1 hour at 4°C to remove DNA, RNA and cell debris. The protein supernatant was precipitated using 5× volumes of precipitation solution (ethanol:acetone:acetic acid at 50:50:0.1 volume ratio) at 4°C overnight, followed by centrifugation. The pellet was dissolved in denaturing buffer (6 M guanidine hydrochloride, 100 mM Tris, cocktail protease inhibitor, pH 8.3) and protein concentration determined by the Bradford assay.

Protein digestion was performed as described elsewhere [37]. Briefly, 600 μg proteins from each sample were treated with DTT (100 μg/1 μl 1 M DTT), alkylated with IAA (100 μg/2 μl 1 M IAA) and ultrafiltered with digestion buffer (50 mM ammonium bicarbonate). The resulting protein solution was incubated with Trypsin (enzyme:protein at 1:40 mass ratio) at 37°C overnight, followed by ultrafiltration and lyophilization. Lyophilized protein samples were then dissolved in loading buffer for the LC-MS/MS analysis.

### 2D LC-MS/MS analysis and peptide identification

Peptide fractionation and analysis was performed in a pH continuous online gradient (pCOG) 2D LC-MS/MS system as described elsewhere [38] with small modifications. Briefly, the peptide solution was loaded on a SCX (Strong Cation Exchange) column (320 μm × 100 mm Column Technology Inc., CA, USA) and eluted resulting in 11 fractions. Each of these fractions was then loaded on two RP (Reversed Phase) alternative trap columns (320 mm, 620 mm, C18, 5 mm, Column Technology) using pH continuous gradient buffer. The following RP gradient was used to elute peptide fractions: 2 to 40% mobile phase (0.1% formic acid (v/v) acetonitrile) in 120 min at 200 *μ*L/min flow rate before the split and 1.5 *μ*L/min after the split. Analysis was performed on the LTQ mass spectrometer equipped with a metal needle electrospray interface mass spectrometer (ThermoFinnigan, San Jose, CA, USA) in a data-dependent collection model (each full scan followed by ten MS/MS scans of most intense ions). All other parameters used were set as described in [39].

The peptides were identified by searching against a combined database of human peptides (IPI human v3.22) and its reversed version using SEQUEST program in BioWorks™ 3.2 software suite. A mass tolerance of 3.0 Da and one missed cleavage site of trypsin were allowed. Cysteine carboxyamidomethlation was set as static modification and no other modification was checked. All output results were filtered and integrated to proteins by an in-house software BuildSummary. At the false discovery rate (FDR) less than 0.5%, all the matches passing a certain Xcorr and delta CN were regarded as valid. Further, all the peptides that could be assigned to multiple proteins were removed. All the protein IDs were mapped to Ensembl gene IDs using Biomart [40]. For each gene, we calculated protein expression level as a median copy number of all peptides mapped to any of the isoforms corresponding to this gene.

### Correlation analysis

To assess the correlation between different platforms, we first set cutoffs to select subsets of genes with discernible expression signals for each platform. Then, we calculated both parametric (Pearson) and non-parametric (Spearman) correlation coefficients between the expression measurements from each pair of platforms using overlapping genes. In the comparison between protein and mRNA expression data, the intersection of genes expressed in all three datasets was used for both microarray/protein and RNA-Seq/protein comparisons to ensure comparability of the obtained correlation values.

For mRNA data, one stringent and one relaxed cutoff were set. For microarrays, the stringent cutoff required expression values of a given gene to pass the detection cutoff (see above) in all samples of the dataset: five individuals or two pooled samples. The relaxed cutoff required expression values in at least one sample of the set to pass the detection cutoff. For RNA sequencing data, the stringent cutoff required at least twenty sequencing reads to map to a given gene in all runs while the relaxed cutoff required at least two reads. For protein expression data, we considered as expressed all 1,037 genes represented by at least two independent peptides identified at FDR < 0.5% (see above). Further, we repeated correlation analysis using 934 and 694 proteins expressed in the firsts and the second set of technical replicates separately (see Additional file 1: Figure S5).

## Authors' contributions

NF, ZY, and CM carried out the experiments. XF, NF, SG, YX, and HH analyzed the data. WC, YL, RZ, and PK conceived of the study, and participated in its design and coordination. All authors helped to draft the manuscript and read and approved the final version.

## Supplementary Material

Additional file 1 **Supplementary figures and tables**. Additional file 1 contains all supplementary figures and tables. Figure S1 – Correlation between gene expression levels measured by Affymetrix arrays. Figure S2 – Correlation between gene expression levels measured by RNA-Seq. Figure S3 – Correlation between gene expression levels measured by Affymetrix arrays and RNA-Seq at different sequencing depth. Figure S4 – Correlation between protein and mRNA expression levels measured by Affymetrix arrays or RNA-Seq using different ways to compute mRNA expression level. Figure S5 – Correlation between mRNA expression levels measured by Affymetrix arrays or RNA-Seq and protein expression levels in two technical replicates. Table S1 – Sample information. Table S2 – Total numbers of sequences in the four sequencing experiments. Table S3 – Correlations between gene expression levels measured by Affymetrix array and RNA-Seq. Table S4 – Correlation between protein expression levels and mRNA expression levels measured by Affymetrix arrays and RNA-Seq. Click here for file
