# Supplementary material for: Estimating accuracy of RNA-Seq and microarrays with proteomics
Source: BMC Genomics. 2009 Apr 16;10:161. doi: 10.1186/1471-2164-10-161 (PMC2676304; doi:10.1186/1471-2164-10-161)
Supplement: Additional file 1 — Supplementary figures and tables. Additional file 1 contains all supplementary figures and tables. Figure S1 – Correlation between gene expression levels measured by Affymetrix arrays. Figure S2 – Correlation between gene expression levels measured by RNA-Seq. Figure S3 – Correlation between gene expression levels measured by Affymetrix arrays and RNA-Seq at different sequencing depth. Figure S4 – Correlation between protein and mRNA expression levels measured by Affymetrix arrays or RNA-Seq using different ways to compute mRNA expression level. Figure S5 – Correlation between mRNA expression levels measured by Affymetrix arrays or RNA-Seq and protein expression levels in two technical replicates. Table S1 – Sample information. Table S2 – Total numbers of sequences in the four sequencing experiments. Table S3 – Correlations between gene expression levels measured by Affymetrix array and RNA-Seq. Table S4 – Correlation between protein expression levels and mRNA expression levels measured by Affymetrix arrays and RNA-Seq. [file 1471-2164-10-161-S1.pdf]

Estimating accuracy of RNA-Seq and microarrays with proteomics

Additional file

Supplementary Figures

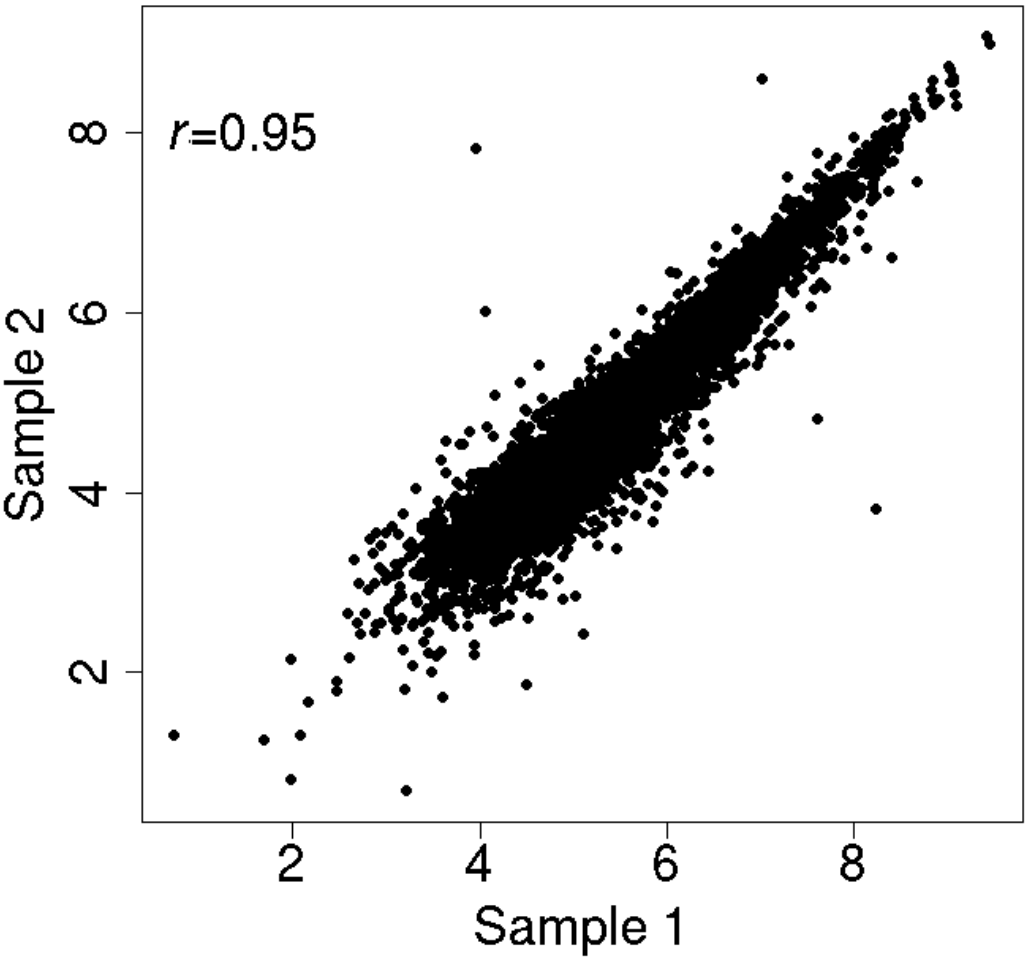

Figure S1 Correlation between gene expression levels measured by Affymetrix arrays. Scatter plot of gene expression levels measured by microarrays in two independent pooled samples.

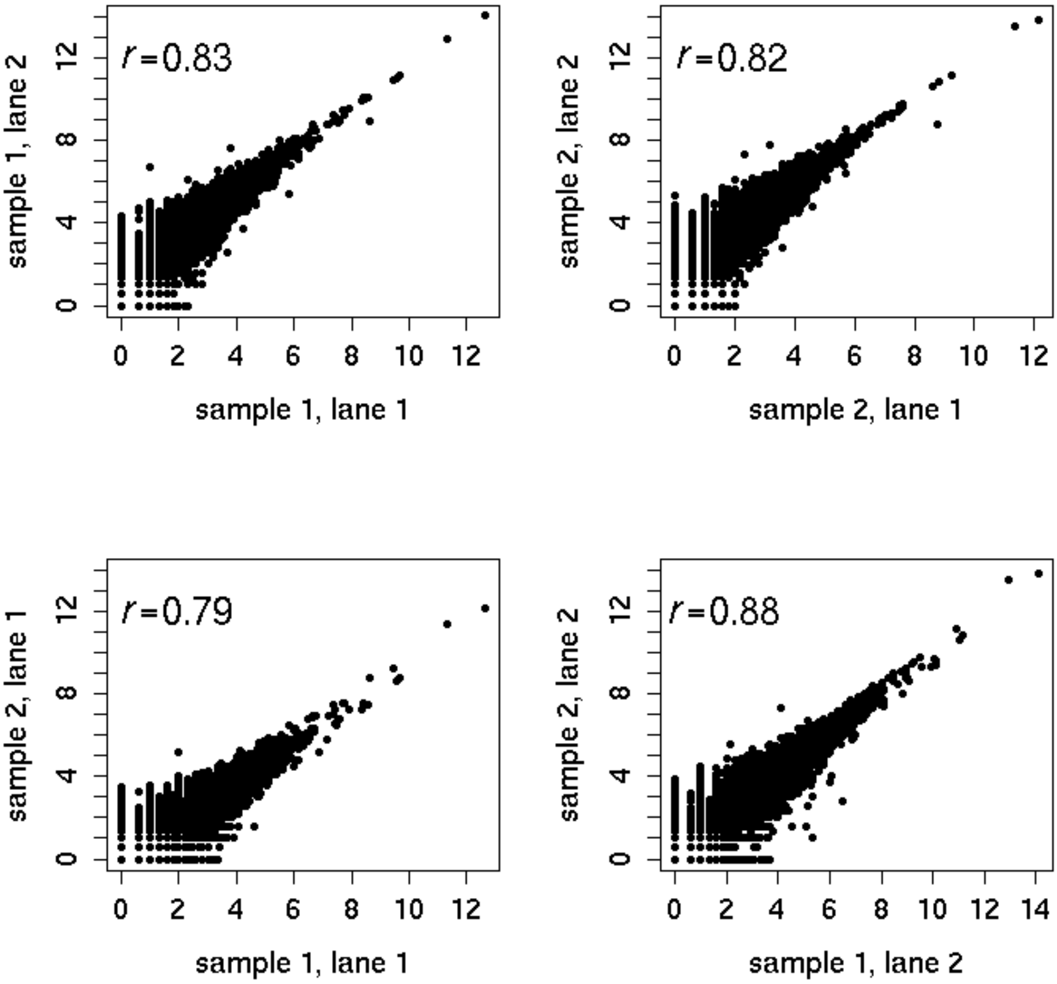

Figure S2 Correlation between gene expression levels measured by RNA-Seq. Scatter plots of gene expression levels measured by RNA-Seq in the biological and technical replicates. Samples 1 and 2 refer to the two independent pooled samples. Lanes 1 and 2 refer to two independent sequencing experiments.

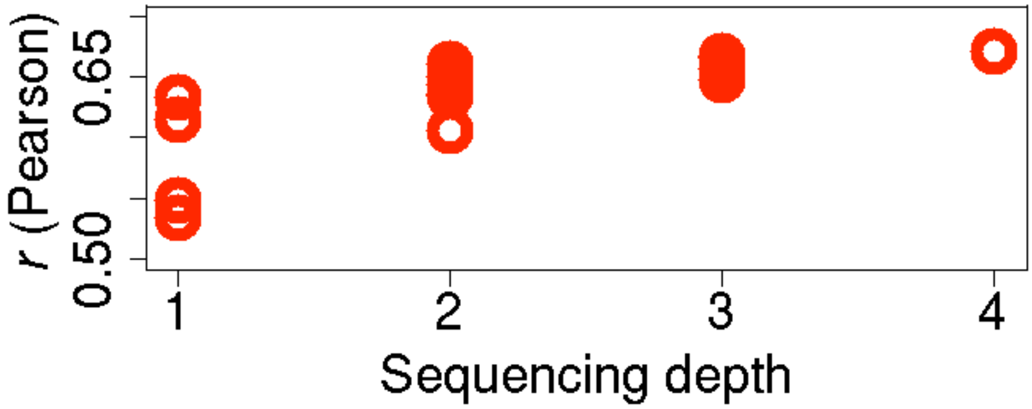

Figure S3 Correlation between gene expression levels measured by Affymetrix arrays and RNA-Seq at different sequencing depth. Pearson correlation coefficients (*r*) from comparisons between microarray and RNA-Seq measurements based on each run separately and on the average expression in all possible combinations of four sequencing runs performed for the two pooled samples. (see Table S3 for details)

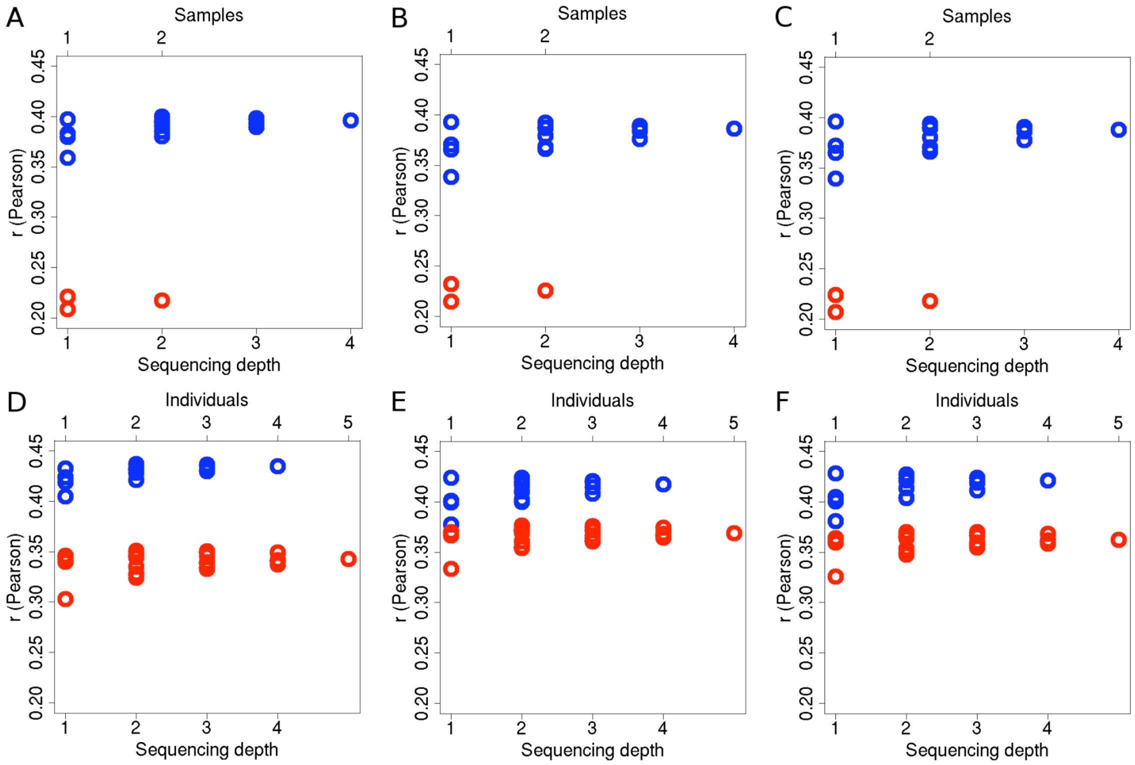

Figure S4 Correlation between protein and mRNA expression levels measured by Affymetrix arrays or RNA-Seq using different ways to compute mRNA expression level. Shown are Person correlation coefficients (*r*) from comparisons between RNA-Seq and protein measurements (blue) and between microarray and protein measurements (red). Protein expression was measured in four individual samples with technical replicates. mRNA expression was measured by microarrays and RNA-Seq in two pooled samples (**A, B, and C**), and by microarrays in 5 individual samples (**D, E, and F**). For RNA-Seq, all exons shared by multiple isoforms were excluded from the analysis. For microarrays, all annotated transcripts did not overlapped. For both RNA-Seq and microarrays, if a gene was represented by multiple transcripts, its expression level was calculated as the maximum (**A and D**), mean (**B and E**), or median (**C and F**) of these transcripts.

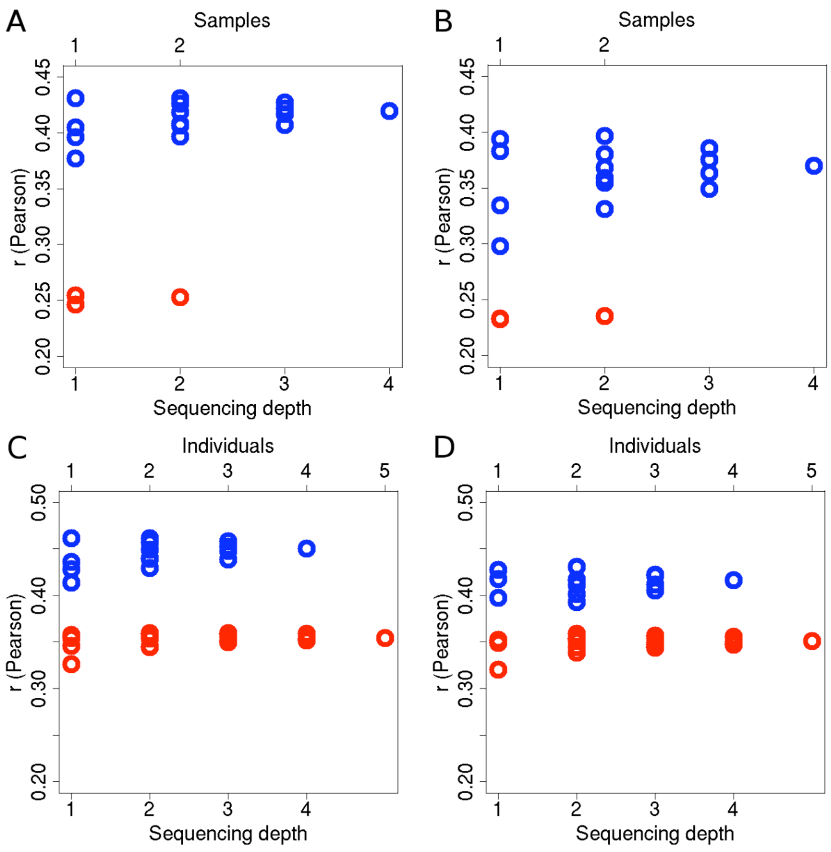

Figure S5 Correlation between mRNA expression levels measured by Affymetrix arrays or RNA-Seq and protein expression levels in two technical replicates. Shown are Person correlation coefficients (*r*) from comparisons between RNA-Seq and protein measurements (blue) and between microarray and protein measurements (red). Protein expression was measured in four individual samples in the first (**A and C**) and in the second (**B and D**) set of technical replicates. mRNA expression was measured by microarrays and RNA-Seq in two pooled samples (**A and B**), and by microarrays in 5 individual samples (**C and D**).

Supplementary Tables

Table S1 Sample information

Sample	Age	Sex	Cause of death	RNA Integrity number from Agilent 2100 bioanalyzer
	8	male	cardiac arrhythmia	7.50
	16	male	accident, multiple injuries	8.40
Human cerebellum pool 1	20	male	accident, lightning striking	8.10
	25	male	asthma	8.00
	53	male	cardiomyopathy	8.60
	16	male	accident, drowning	7.70
	12	male	asthma	6.80
Human cerebellum pool 2	21	male	accident, multiple injuries	7.60
	27	male	accident, multiple injuries	7.80
	54	male	ASCVD	7.20
Human cerebellum individual 1	39	male	HASCVD	8.70
Human cerebellum individual 2	45	male	accident	8.10
Human cerebellum individual 3	45	male	accident	7.90
Human cerebellum individual 4	59	male	cardiomyopathy	7.30
Human cerebellum individual 5	71	male	IAA	6.70

Note: Highlighted individuals were used for both microarray and proteomics experiments.

Table S2 Total numbers of sequences in the four sequencing experiments.

	Sample 1	Sample 2
Run 1	2,633,175	2,063,591
Run 2	6,709,345	5,245,797

Table S3 Correlations between gene expression levels measured by Affymetrix array and RNA-Seq.

Affymetrix	RNA-Seq	Number of genes	Sample/individual/run	Spearman		Pearson	
				r	*p*	*r*	*p*
>= 1 sample	>= 2 reads	8441	sample 1	0.65	0	0.66	0
>= 1 sample	>= 2 reads	8441	sample 2	0.65	0	0.66	0
>= 1 sample	>= 2 reads	8441	average 2 samples	0.66	0	0.67	0
>= 1 sample	>= 20 reads	4574	sample 1	0.54	0	0.55	0
>= 1 sample	>= 20 reads	4574	sample 2	0.55	0	0.57	0
>= 1 sample	>= 20 reads	4574	average 2 samples	0.55	0	0.57	0
2 samples	>= 2 reads	6327	sample 1	0.56	0	0.58	0
2 samples	>= 2 reads	6327	sample 2	0.55	0	0.57	0
2 samples	>= 2 reads	6327	average 2 samples	0.56	0	0.58	0
2 samples	>= 20 reads	4181	sample 1	0.52	1.2E-284	0.54	0

2 samples	>= 20 reads	4181	sample 2	0.53	1.4E-302	0.55	0
2 samples	>= 20 reads	4181	average 2 samples	0.53	2E-300	0.55	0
>= 1 sample	>= 2 reads	8441	run 1	0.52	0	0.55	0
>= 1 sample	>= 2 reads	8441	run 2	0.51	0	0.53	0
>= 1 sample	>= 2 reads	8441	run 3	0.61	0	0.62	0
>= 1 sample	>= 2 reads	8441	run 4	0.62	0	0.63	0
>= 1 sample	>= 2 reads	8441	average 2 runs	0.59	0	0.61	0
>= 1 sample	>= 2 reads	8441	average 2 runs	0.63	0	0.63	0
>= 1 sample	>= 2 reads	8441	average 2 runs	0.64	0	0.65	0
>= 1 sample	>= 2 reads	8441	average 2 runs	0.63	0	0.64	0
>= 1 sample	>= 2 reads	8441	average 2 runs	0.64	0	0.64	0
>= 1 sample	>= 2 reads	8441	average 2 runs	0.65	0	0.66	0
>= 1 sample	>= 2 reads	8441	average 3 runs	0.64	0	0.65	0
>= 1 sample	>= 2 reads	8441	average 3 runs	0.65	0	0.66	0
>= 1 sample	>= 2 reads	8441	average 3 runs	0.66	0	0.67	0
>= 1 sample	>= 2 reads	8441	average 3 runs	0.66	0	0.67	0
>= 1 sample	>= 2 reads	8441	average 4 runs	0.66	0	0.67	0
>=1 individual	>=2 reads	4758	individual 1	0.59	0	0.62	0
>=1 individual	>=2 reads	4758	individual 2	0.61	0	0.64	0
>=1 individual	>=2 reads	4758	individual 3	0.55	0	0.58	0
>=1 individual	>=2 reads	4758	individual 4	0.59	0	0.61	0
>=1 individual	>=2 reads	4758	individual 5	0.54	0	0.56	0
>=1 individual	>=2 reads	4758	average 2 individuals	0.61	0	0.63	0
>=1 individual	>=2 reads	4758	average 2 individuals	0.58	0	0.60	0
>=1 individual	>=2 reads	4758	average 2 individuals	0.59	0	0.62	0
>=1 individual	>=2 reads	4758	average 2 individuals	0.57	0	0.60	0
>=1 individual	>=2 reads	4758	average 2 individuals	0.59	0	0.62	0
>=1 individual	>=2 reads	4758	average 2 individuals	0.60	0	0.63	0
>=1 individual	>=2 reads	4758	average 2 individuals	0.59	0	0.61	0
>=1 individual	>=2 reads	4758	average 2 individuals	0.57	0	0.60	0
>=1 individual	>=2 reads	4758	average 2 individuals	0.55	0	0.57	0
>=1 individual	>=2 reads	4758	average 2 individuals	0.57	0	0.59	0
>=1 individual	>=2 reads	4758	average 3 individuals	0.59	0	0.62	0
>=1 individual	>=2 reads	4758	average 3 individuals	0.60	0	0.63	0
>=1 individual	>=2 reads	4758	average 3 individuals	0.59	0	0.62	0
>=1 individual	>=2 reads	4758	average 3 individuals	0.58	0	0.61	0
>=1 individual	>=2 reads	4758	average 3 individuals	0.57	0	0.59	0
>=1 individual	>=2 reads	4758	average 3 individuals	0.58	0	0.61	0
>=1 individual	>=2 reads	4758	average 3 individuals	0.59	0	0.62	0
>=1 individual	>=2 reads	4758	average 3 individuals	0.58	0	0.60	0
>=1 individual	>=2 reads	4758	average 3 individuals	0.59	0	0.61	0
>=1 individual	>=2 reads	4758	average 3 individuals	0.56	0	0.59	0
>=1 individual	>=2 reads	4758	average 4 individuals	0.59	0	0.62	0
>=1 individual	>=2 reads	4758	average 4 individuals	0.58	0	0.61	0
>=1 individual	>=2 reads	4758	average 4 individuals	0.59	0	0.62	0
>=1 individual	>=2 reads	4758	average 4 individuals	0.57	0	0.60	0

>=1 individual	>=2 reads	4758	average 4 individuals	0.58	0	0.61	0
>=1 individual	>=2 reads	4758	average 5 individuals	0.59	0	0.61	0
5 individuals	>=2 reads	2655	individual 1	0.57	8.2E-225	0.59	0
5 individuals	>=2 reads	2655	individual 2	0.58	2E-243	0.61	0
5 individuals	>=2 reads	2655	individual 3	0.53	7.9E-193	0.56	0
5 individuals	>=2 reads	2655	individual 4	0.56	3.1E-222	0.59	0
5 individuals	>=2 reads	2655	individual 5	0.52	1.1E-187	0.55	0
5 individuals	>=2 reads	2655	average 2 individuals	0.58	2E-240	0.61	0
5 individuals	>=2 reads	2655	average 2 individuals	0.56	1.2E-214	0.58	0
5 individuals	>=2 reads	2655	average 2 individuals	0.57	8.8E-227	0.59	0
5 individuals	>=2 reads	2655	average 2 individuals	0.55	1.4E-212	0.58	0
5 individuals	>=2 reads	2655	average 2 individuals	0.57	9.6E-228	0.60	0
5 individuals	>=2 reads	2655	average 2 individuals	0.58	2.2E-237	0.60	0
5 individuals	>=2 reads	2655	average 2 individuals	0.57	3.9E-224	0.59	0
5 individuals	>=2 reads	2655	average 2 individuals	0.55	1.5E-212	0.58	0
5 individuals	>=2 reads	2655	average 2 individuals	0.53	2.6E-193	0.56	0
5 individuals	>=2 reads	2655	average 2 individuals	0.55	1.7E-209	0.58	0
5 individuals	>=2 reads	2655	average 3 individuals	0.57	2.7E-230	0.60	0
5 individuals	>=2 reads	2655	average 3 individuals	0.58	1.6E-236	0.60	0
5 individuals	>=2 reads	2655	average 3 individuals	0.57	3.5E-228	0.59	0
5 individuals	>=2 reads	2655	average 3 individuals	0.56	1.5E-219	0.59	0
5 individuals	>=2 reads	2655	average 3 individuals	0.55	1.1E-208	0.58	0
5 individuals	>=2 reads	2655	average 3 individuals	0.56	5.3E-218	0.58	0
5 individuals	>=2 reads	2655	average 3 individuals	0.57	5.7E-228	0.59	0
5 individuals	>=2 reads	2655	average 3 individuals	0.56	5.3E-217	0.58	0
5 individuals	>=2 reads	2655	average 3 individuals	0.57	1E-225	0.59	0
5 individuals	>=2 reads	2655	average 3 individuals	0.55	2E-206	0.57	0
5 individuals	>=2 reads	2655	average 4 individuals	0.57	1.7E-229	0.60	0
5 individuals	>=2 reads	2655	average 4 individuals	0.56	3.2E-222	0.59	0
5 individuals	>=2 reads	2655	average 4 individuals	0.57	5.6E-228	0.59	0
5 individuals	>=2 reads	2655	average 4 individuals	0.55	4.2E-214	0.58	0
5 individuals	>=2 reads	2655	average 4 individuals	0.56	2.1E-220	0.59	0
5 individuals	>=2 reads	2655	average 5 individuals	0.56	2E-223	0.59	0
>=1 individual	>=20 reads	3253	individual 1	0.57	6.4E-276	0.61	0
>=1 individual	>=20 reads	3253	individual 2	0.58	4.3E-291	0.62	0
>=1 individual	>=20 reads	3253	individual 3	0.51	1.7E-215	0.56	0
>=1 individual	>=20 reads	3253	individual 4	0.55	8.1E-262	0.59	0
>=1 individual	>=20 reads	3253	individual 5	0.51	2.1E-219	0.56	0
>=1 individual	>=20 reads	3253	average 2 individuals	0.58	2E-291	0.62	0
>=1 individual	>=20 reads	3253	average 2 individuals	0.54	4.1E-250	0.59	0
>=1 individual	>=20 reads	3253	average 2 individuals	0.56	5.7E-272	0.60	0
>=1 individual	>=20 reads	3253	average 2 individuals	0.55	6.5E-254	0.59	0
>=1 individual	>=20 reads	3253	average 2 individuals	0.56	2.3E-264	0.60	0
>=1 individual	>=20 reads	3253	average 2 individuals	0.57	8.9E-282	0.61	0
>=1 individual	>=20 reads	3253	average 2 individuals	0.56	1.3E-266	0.60	0
>=1 individual	>=20 reads	3253	average 2 individuals	0.54	2.4E-243	0.58	0

>=1 individual	>=20 reads	3253	average 2 individuals	0.52	2.5E-221	0.56	0
>=1 individual	>=20 reads	3253	average 2 individuals	0.54	3.1E-246	0.58	0
>=1 individual	>=20 reads	3253	average 3 individuals	0.56	1.8E-271	0.61	0
>=1 individual	>=20 reads	3253	average 3 individuals	0.57	1.3E-283	0.61	0
>=1 individual	>=20 reads	3253	average 3 individuals	0.57	7.8E-274	0.61	0
>=1 individual	>=20 reads	3253	average 3 individuals	0.55	9.3E-257	0.59	0
>=1 individual	>=20 reads	3253	average 3 individuals	0.54	1.9E-243	0.58	0
>=1 individual	>=20 reads	3253	average 3 individuals	0.55	2.1E-259	0.59	0
>=1 individual	>=20 reads	3253	average 3 individuals	0.56	1.1E-265	0.60	0
>=1 individual	>=20 reads	3253	average 3 individuals	0.55	2E-253	0.59	0
>=1 individual	>=20 reads	3253	average 3 individuals	0.56	8.7E-268	0.60	0
>=1 individual	>=20 reads	3253	average 3 individuals	0.53	1.2E-238	0.58	0
>=1 individual	>=20 reads	3253	average 4 individuals	0.56	1.9E-270	0.60	0
>=1 individual	>=20 reads	3253	average 4 individuals	0.55	4.3E-262	0.60	0
>=1 individual	>=20 reads	3253	average 4 individuals	0.56	2.6E-272	0.60	0
>=1 individual	>=20 reads	3253	average 4 individuals	0.54	1.6E-250	0.59	0
>=1 individual	>=20 reads	3253	average 4 individuals	0.55	8.5E-258	0.59	0
>=1 individual	>=20 reads	3253	average 5 individuals	0.56	2.3E-263	0.60	0
5 individuals	>=20 reads	2159	individual 1	0.60	4.5E-207	0.63	0
5 individuals	>=20 reads	2159	individual 2	0.60	6.7E-214	0.64	0
5 individuals	>=20 reads	2159	individual 3	0.54	5.4E-167	0.59	0
5 individuals	>=20 reads	2159	individual 4	0.58	1.1E-196	0.62	0
5 individuals	>=20 reads	2159	individual 5	0.55	3.1E-168	0.59	0
5 individuals	>=20 reads	2159	average 2 individuals	0.61	1.6E-216	0.64	0
5 individuals	>=20 reads	2159	average 2 individuals	0.58	7.6E-192	0.62	0
5 individuals	>=20 reads	2159	average 2 individuals	0.59	1.1E-204	0.63	0
5 individuals	>=20 reads	2159	average 2 individuals	0.58	1.8E-193	0.62	0
5 individuals	>=20 reads	2159	average 2 individuals	0.59	1.3E-199	0.63	0
5 individuals	>=20 reads	2159	average 2 individuals	0.60	8.6E-210	0.64	0
5 individuals	>=20 reads	2159	average 2 individuals	0.59	8.2E-200	0.63	0
5 individuals	>=20 reads	2159	average 2 individuals	0.57	4.7E-186	0.61	0
5 individuals	>=20 reads	2159	average 2 individuals	0.55	1.6E-170	0.60	0
5 individuals	>=20 reads	2159	average 2 individuals	0.57	8.8E-187	0.61	0
5 individuals	>=20 reads	2159	average 3 individuals	0.59	4.6E-205	0.63	0
5 individuals	>=20 reads	2159	average 3 individuals	0.60	9E-212	0.64	0
5 individuals	>=20 reads	2159	average 3 individuals	0.59	1.6E-205	0.63	0
5 individuals	>=20 reads	2159	average 3 individuals	0.58	1.1E-195	0.62	0
5 individuals	>=20 reads	2159	average 3 individuals	0.57	8.5E-187	0.61	0
5 individuals	>=20 reads	2159	average 3 individuals	0.58	1.5E-196	0.62	0
5 individuals	>=20 reads	2159	average 3 individuals	0.59	1.9E-200	0.63	0
5 individuals	>=20 reads	2159	average 3 individuals	0.58	8E-192	0.62	0
5 individuals	>=20 reads	2159	average 3 individuals	0.59	9.3E-201	0.63	0
5 individuals	>=20 reads	2159	average 3 individuals	0.57	2.3E-182	0.61	0
5 individuals	>=20 reads	2159	average 4 individuals	0.59	4.4E-204	0.63	0
5 individuals	>=20 reads	2159	average 4 individuals	0.58	2.1E-198	0.62	0
5 individuals	>=20 reads	2159	average 4 individuals	0.59	2.6E-204	0.63	0

5 individuals	>=20 reads	2159	average 4 individuals	0.58	4E-191	0.62	0
5 individuals	>=20 reads	2159	average 4 individuals	0.58	7.3E-195	0.62	0
5 individuals	>=20 reads	2159	average 5 individuals	0.59	3.7E-199	0.62	0

Table S4 Correlation between protein expression levels and mRNA expression levels measured by Affymetrix arrays and RNA-Seq.

Platform	Cutoff	Number of genes	Sample/individual/run	Spearman		Pearson	
				r	*p*	*r*	*p*
Affymetrix	>=1 sample	800	sample 1	0.21	4.04E-09	0.24	9.13E-12
Affymetrix	>=1 sample	800	sample 2	0.20	5.94E-09	0.24	6.33E-12
Affymetrix	>=1 sample	800	average 2 samples	0.21	3.37E-09	0.24	4.43E-12
Affymetrix	2 samples	520	sample 1	0.20	3.93E-06	0.23	8.05E-08
Affymetrix	2 samples	520	sample 2	0.20	2.44E-06	0.24	1.64E-08
Affymetrix	2 samples	520	average 2 samples	0.21	2.12E-06	0.24	2.66E-08
RNA-Seq	>=2 reads	800	run 1	0.18	4.3E-07	0.30	0
RNA-Seq	>=2 reads	800	run 2	0.19	9.59E-08	0.29	0
RNA-Seq	>=2 reads	800	run 3	0.22	1.73E-10	0.32	0
RNA-Seq	>=2 reads	800	run 4	0.26	2.39E-13	0.31	0
RNA-Seq	>=2 reads	800	average 2 runs	0.20	9.55E-09	0.31	0
RNA-Seq	>=2 reads	800	average 2 runs	0.22	1.85E-10	0.32	0
RNA-Seq	>=2 reads	800	average 2 runs	0.25	5.09E-13	0.32	0
RNA-Seq	>=2 reads	800	average 2 runs	0.23	5.24E-11	0.32	0
RNA-Seq	>=2 reads	800	average 2 runs	0.25	2.96E-13	0.31	0
RNA-Seq	>=2 reads	800	average 2 runs	0.25	6.6E-13	0.32	0
RNA-Seq	>=2 reads	800	average 3 runs	0.23	6.32E-11	0.32	0
RNA-Seq	>=2 reads	800	average 3 runs	0.25	5.66E-13	0.31	0
RNA-Seq	>=2 reads	800	average 3 runs	0.25	1.21E-12	0.32	0
RNA-Seq	>=2 reads	800	average 3 runs	0.25	8.07E-13	0.32	0
RNA-Seq	>=2 reads	800	average 4 runs	0.25	1.36E-12	0.32	0
RNA-Seq	>=20 reads	520	run 1	0.22	2.35E-07	0.34	2.22E-15
RNA-Seq	>=20 reads	520	run 2	0.21	9.23E-07	0.32	6.31E-14
RNA-Seq	>=20 reads	520	run 3	0.29	1.79E-11	0.36	0
RNA-Seq	>=20 reads	520	run 4	0.29	1.5E-11	0.36	0
RNA-Seq	>=20 reads	520	average 2 runs	0.24	2.24E-08	0.34	1.55E-15
RNA-Seq	>=20 reads	520	average 2 runs	0.29	2.64E-11	0.36	0
RNA-Seq	>=20 reads	520	average 2 runs	0.29	7.46E-12	0.36	0
RNA-Seq	>=20 reads	520	average 2 runs	0.29	1.51E-11	0.36	0
RNA-Seq	>=20 reads	520	average 2 runs	0.29	1.52E-11	0.35	2.22E-16
RNA-Seq	>=20 reads	520	average 2 runs	0.30	1.44E-12	0.36	0
RNA-Seq	>=20 reads	520	average 3 runs	0.29	2.05E-11	0.36	0
RNA-Seq	>=20 reads	520	average 3 runs	0.29	1.1E-11	0.35	0
RNA-Seq	>=20 reads	520	average 3 runs	0.30	1.75E-12	0.36	0
RNA-Seq	>=20 reads	520	average 3 runs	0.30	2.45E-12	0.36	0
RNA-Seq	>=20 reads	520	average 4 runs	0.30	2.84E-12	0.36	0
Affymetrix	>=1 individual	565	individual 1	0.26	1.8E-10	0.30	1.49E-13
Affymetrix	>=1 individual	565	individual 2	0.23	2.96E-08	0.28	2.12E-11

Affymetrix	>=1 individual	565	individual 3	0.24	7.92E-09	0.28	1.42E-11
Affymetrix	>=1 individual	565	individual 4	0.25	2.66E-09	0.29	1.13E-12
Affymetrix	>=1 individual	565	individual 5	0.27	3.06E-11	0.31	3.49E-14
Affymetrix	>=1 individual	565	average 2 individuals	0.25	2.43E-09	0.29	1.18E-12
Affymetrix	>=1 individual	565	average 2 individuals	0.25	8.37E-10	0.29	8.49E-13
Affymetrix	>=1 individual	565	average 2 individuals	0.26	6.8E-10	0.30	3.28E-13
Affymetrix	>=1 individual	565	average 2 individuals	0.27	4.78E-11	0.31	3.51E-14
Affymetrix	>=1 individual	565	average 2 individuals	0.24	6.12E-09	0.28	5.6E-12
Affymetrix	>=1 individual	565	average 2 individuals	0.24	7.68E-09	0.29	3.31E-12
Affymetrix	>=1 individual	565	average 2 individuals	0.26	6.64E-10	0.30	3.04E-13
Affymetrix	>=1 individual	565	average 2 individuals	0.25	3.29E-09	0.29	2.33E-12
Affymetrix	>=1 individual	565	average 2 individuals	0.26	4.81E-10	0.30	4.79E-13
Affymetrix	>=1 individual	565	average 2 individuals	0.26	2.19E-10	0.31	1.13E-13
Affymetrix	>=1 individual	565	average 3 individuals	0.25	1.72E-09	0.29	1.37E-12
Affymetrix	>=1 individual	565	average 3 individuals	0.25	2.07E-09	0.29	9.59E-13
Affymetrix	>=1 individual	565	average 3 individuals	0.26	3.24E-10	0.30	1.81E-13
Affymetrix	>=1 individual	565	average 3 individuals	0.25	1.11E-09	0.30	7.45E-13
Affymetrix	>=1 individual	565	average 3 individuals	0.26	2.12E-10	0.30	2.02E-13
Affymetrix	>=1 individual	565	average 3 individuals	0.26	1.67E-10	0.31	9.3E-14
Affymetrix	>=1 individual	565	average 3 individuals	0.24	4.1E-09	0.29	2.75E-12
Affymetrix	>=1 individual	565	average 3 individuals	0.25	1.01E-09	0.30	6.75E-13
Affymetrix	>=1 individual	565	average 3 individuals	0.25	9.56E-10	0.30	3.89E-13
Affymetrix	>=1 individual	565	average 3 individuals	0.26	6.14E-10	0.30	4.23E-13
Affymetrix	>=1 individual	565	average 4 individuals	0.25	1.91E-09	0.29	1.16E-12
Affymetrix	>=1 individual	565	average 4 individuals	0.26	4.65E-10	0.30	3.76E-13
Affymetrix	>=1 individual	565	average 4 individuals	0.26	5.37E-10	0.30	2.56E-13
Affymetrix	>=1 individual	565	average 4 individuals	0.26	3.79E-10	0.30	2.55E-13
Affymetrix	>=1 individual	565	average 4 individuals	0.25	1.08E-09	0.30	6.58E-13
Affymetrix	>=1 individual	565	average 5 individuals	0.26	6.99E-10	0.30	4.23E-13
Affymetrix	5 individuals	306	individual 1	0.30	7.7E-08	0.34	5.83E-10
Affymetrix	5 individuals	306	individual 2	0.29	2.05E-07	0.34	6.52E-10
Affymetrix	5 individuals	306	individual 3	0.27	2.5E-06	0.30	5.29E-08
Affymetrix	5 individuals	306	individual 4	0.29	2.98E-07	0.34	1.73E-09
Affymetrix	5 individuals	306	individual 5	0.30	1.33E-07	0.34	1.66E-09
Affymetrix	5 individuals	306	average 2 individuals	0.30	9.81E-08	0.35	3.9E-10
Affymetrix	5 individuals	306	average 2 individuals	0.28	4.34E-07	0.33	3.92E-09
Affymetrix	5 individuals	306	average 2 individuals	0.30	1.43E-07	0.34	8.37E-10
Affymetrix	5 individuals	306	average 2 individuals	0.30	1.15E-07	0.34	6.74E-10
Affymetrix	5 individuals	306	average 2 individuals	0.29	2.66E-07	0.33	2.22E-09
Affymetrix	5 individuals	306	average 2 individuals	0.29	2E-07	0.34	7.31E-10
Affymetrix	5 individuals	306	average 2 individuals	0.30	1.12E-07	0.35	4.61E-10
Affymetrix	5 individuals	306	average 2 individuals	0.28	8.29E-07	0.32	6.67E-09
Affymetrix	5 individuals	306	average 2 individuals	0.28	5.95E-07	0.32	7.43E-09
Affymetrix	5 individuals	306	average 2 individuals	0.29	1.88E-07	0.34	1.25E-09
Affymetrix	5 individuals	306	average 3 individuals	0.29	1.75E-07	0.34	1.21E-09
Affymetrix	5 individuals	306	average 3 individuals	0.30	1.26E-07	0.35	5.52E-10

Affymetrix	5 individuals	306	average 3 individuals	0.30	8.87E-08	0.35	4.07E-10
Affymetrix	5 individuals	306	average 3 individuals	0.29	3.77E-07	0.33	2.51E-09
Affymetrix	5 individuals	306	average 3 individuals	0.29	2.7E-07	0.33	2.42E-09
Affymetrix	5 individuals	306	average 3 individuals	0.30	1.45E-07	0.34	8.07E-10
Affymetrix	5 individuals	306	average 3 individuals	0.29	2.64E-07	0.34	1.81E-09
Affymetrix	5 individuals	306	average 3 individuals	0.29	2.18E-07	0.34	1.57E-09
Affymetrix	5 individuals	306	average 3 individuals	0.30	1.35E-07	0.34	6.34E-10
Affymetrix	5 individuals	306	average 3 individuals	0.28	4.64E-07	0.33	3.57E-09
Affymetrix	5 individuals	306	average 4 individuals	0.29	2.05E-07	0.34	1.22E-09
Affymetrix	5 individuals	306	average 4 individuals	0.29	1.5E-07	0.34	1.06E-09
Affymetrix	5 individuals	306	average 4 individuals	0.30	1.12E-07	0.35	5.42E-10
Affymetrix	5 individuals	306	average 4 individuals	0.29	2.91E-07	0.33	1.94E-09
Affymetrix	5 individuals	306	average 4 individuals	0.29	1.98E-07	0.34	1.46E-09
Affymetrix	5 individuals	306	average 5 individuals	0.29	1.68E-07	0.34	1.09E-09
RNA-Seq	>=2 reads	565	run 1	0.14	0.001154	0.32	6.22E-15
RNA-Seq	>=2 reads	565	run 2	0.16	0.000199	0.32	1.31E-14
RNA-Seq	>=2 reads	565	run 3	0.19	5.44E-06	0.34	0
RNA-Seq	>=2 reads	565	run 4	0.23	6.17E-08	0.34	0
RNA-Seq	>=2 reads	565	average 2 runs	0.16	0.000113	0.33	1.11E-15
RNA-Seq	>=2 reads	565	average 2 runs	0.18	1.42E-05	0.34	0
RNA-Seq	>=2 reads	565	average 2 runs	0.22	1.9E-07	0.34	0
RNA-Seq	>=2 reads	565	average 2 runs	0.19	4.9E-06	0.34	0
RNA-Seq	>=2 reads	565	average 2 runs	0.22	1.29E-07	0.34	0
RNA-Seq	>=2 reads	565	average 2 runs	0.22	1.71E-07	0.35	0
RNA-Seq	>=2 reads	565	average 3 runs	0.19	9.38E-06	0.34	0
RNA-Seq	>=2 reads	565	average 3 runs	0.21	2.95E-07	0.34	0
RNA-Seq	>=2 reads	565	average 3 runs	0.21	3.98E-07	0.35	0
RNA-Seq	>=2 reads	565	average 3 runs	0.21	3.01E-07	0.35	0
RNA-Seq	>=2 reads	565	average 4 runs	0.21	5.45E-07	0.35	0
RNA-Seq	>=20 reads	306	run 1	0.24	1.49E-05	0.40	2.64E-13
RNA-Seq	>=20 reads	306	run 2	0.28	4.78E-07	0.39	1.48E-12
RNA-Seq	>=20 reads	306	run 3	0.31	2.44E-08	0.42	7.55E-15
RNA-Seq	>=20 reads	306	run 4	0.31	2.35E-08	0.41	5.06E-14
RNA-Seq	>=20 reads	306	average 2 runs	0.28	7.9E-07	0.41	1.27E-13
RNA-Seq	>=20 reads	306	average 2 runs	0.30	6.37E-08	0.42	8.88E-15
RNA-Seq	>=20 reads	306	average 2 runs	0.31	2.48E-08	0.42	2.86E-14
RNA-Seq	>=20 reads	306	average 2 runs	0.32	1.49E-08	0.42	1.29E-14
RNA-Seq	>=20 reads	306	average 2 runs	0.32	1.66E-08	0.41	7.11E-14
RNA-Seq	>=20 reads	306	average 2 runs	0.32	6.18E-09	0.42	1.15E-14
RNA-Seq	>=20 reads	306	average 3 runs	0.31	3.12E-08	0.42	1.27E-14
RNA-Seq	>=20 reads	306	average 3 runs	0.31	1.94E-08	0.41	3.95E-14
RNA-Seq	>=20 reads	306	average 3 runs	0.32	1.03E-08	0.42	1.09E-14
RNA-Seq	>=20 reads	306	average 3 runs	0.32	7.31E-09	0.42	1.64E-14
RNA-Seq	>=20 reads	306	average 4 runs	0.32	1.04E-08	0.42	1.44E-14
